# Novel Acaricidal Drug Fluazuron Causes Immunotoxicity via Selective Depletion of Lymphocytes T CD8

**DOI:** 10.1155/2019/2815461

**Published:** 2019-05-07

**Authors:** Juliana Gonçalves Ribeiro, Anelise Santos Soares, Pamella Eduardha Espindola Chaves, Jéssica Tamara Limberger, Emanoeli da Rosa, Luísa Zuravski, Luís Flávio Souza de Oliveira, Michel Mansur Machado

**Affiliations:** ^1^Postgraduate Program in Pharmaceutical Sciences, Federal University of Pampa, BR 472, Km 585, Mailbox 118, CEP: 97500-970, Uruguaiana, RS, Brazil; ^2^TOXCEL-Cellular Toxicology Research Group, Federal University of Pampa, BR 472, Km 585, Mailbox 118, CEP: 97500-970, Uruguaiana, RS, Brazil

## Abstract

Fluazuron is one of the newest veterinary antitick medicines. Belonging to the benzoylphenylureas group, its mechanism of action acts by the interference of the formation of the chitin of the tick, which is responsible for the hardening of its exoskeletons. In addition to taking care of the health of the animal so that it receives the medication in the doses and the correct form, it is important to analyze the safety of the operator. Reduced resistance to infectious disease was a well-documented consequence of primary and acquired immunodeficiencies, but a novel finding following xenobiotic exposure. The awareness of the consequences of altered immune function is the most likely outcome of inadvertent exposure. The human health implications of studies in which chemical exposure reduced resistance to infection drove an early focus on immunosuppression within the toxicology community. The main objective is to perform the evaluation by computational platforms and in cell culture, searching for data that can serve as a foundation for a better understanding of the toxic effects involved with the accidental contamination of Fluazuron and, thus, to assist the medical community and users to understand the risks inherent in its use. As far as we can determine in the literature, our work has unmistakably demonstrated that the Fluazuron can cause genotoxicity by probable chromatin rearrangement and immunodepleting by specific reduction of the CD8 T lymphocyte subpopulation, mediated by the decrease in gamma interferon production. Although the use of Fluazuron is a necessity for tick control and for cattle management, we must bear in mind that the imminent risks to its application exist. Careless use can damage the immune system which in turn carries a gigantic hazard by opening a door to diseases and pathogens and leaving us defenseless.

## 1. Introduction

In recent years there have been several changes in beef cattle, with modern production applications, which allowed the sector to increase volume and productivity [1]. Due to the increase in animal production, there was a multiplication of the cases of parasitism, developing a wide range of parasitic species, one among them being the tick* Rhipicephalus (Boophilus) microplus *[2].

One of the main control methods for the tick is the use of acaricides, the main ones being applied under immersion, spray, pour-on, and injectable formulations and the latter two being classified as systemic action [3].

**Figure 1:**
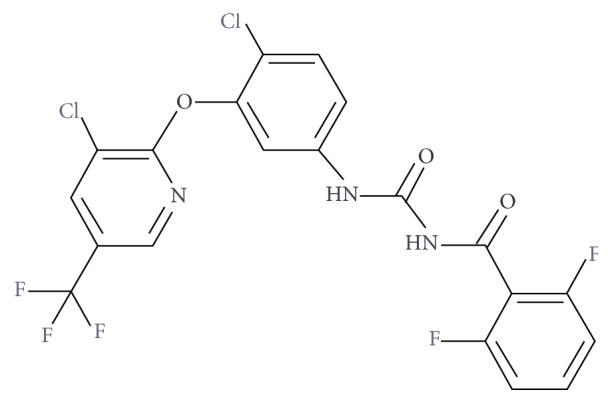
Fluazuron chemical structure [9].

In the market there are several active principles and formulations. Fluazuron (N-[[4-chloro-3-[3-chloro-5-(trifluoromethyl)pyridin-2-yl]oxyphenyl]carbamoyl]-2,6-difluorobenzamide) (Figure 1) is one of the newest veterinary antitick medicines, which has the “pour-on” formula in which it means to apply the drug along the animal's back [4]. Belonging to the benzoylphenylureas group, its mechanism of action acts by the interference of the formation of the chitin of the tick, which is responsible for the hardening of its exoskeletons [5]. After administration of Fluazuron, absorption occurs slowly, and elimination can be observed for 3-4 weeks after treatment. Fluazuron was not extensively metabolized as unchanged fluazuron accounted for more than 90% of the total [6].

In addition to taking care of the health of the animal so that it receives the medication in the doses and the correct form, it is important to analyze the safety of the operator. Carrapaticides are poisons that act primarily on the central nervous system (CNS), causing allergies, malformations of organs, tumor processes, and mainly intoxications. Typically, individuals who have contact with these products are the same on the property and, as they often do the handling, tend to decrease care with these toxic substances [7].

Reduced resistance to infectious disease was a well-documented consequence of primary and acquired immunodeficiencies, but a novel finding following xenobiotic exposure. The awareness of the consequences of altered immune function is the most likely outcome of inadvertent exposure. The human health implications of studies in which chemical exposure reduced resistance to infection drove an early focus on immunosuppression within the toxicology community [12].

Currently there are few available studies on their toxicity to humans, mainly in relation to low concentrations and risks of genetic toxicity. Because of this, an alternative means of performing the first steps of a research would be the in silico and* in vitro* models.

In silico computational models are developed using several programs that allow the prediction of the risk and danger of various chemical substances according to their molecular structure. There is now a wide range of free software available to predict chemical properties, toxicological parameters, and other effects [13]. For many years,* in vitro* models have been used for several tests during the research. They are effective in replacing tests on animals that are limited in time and ethical aspects, in addition to the financial burden [14].

In our study, the main objective is to perform the evaluation by computational platforms and in cell culture, searching for data that can serve as a foundation for a better understanding of the toxic effects involved with the accidental contamination of Fluazuron and, thus, to assist the medical community and users to understand the risks inherent in its use.

## 2. Material and Methods

### 2.1. Chemical

All chemicals were of analytical grade and were acquired from Sigma Chemical Co. (St. Louis, MO, USA).

### 2.2. Peripheral Blood Mononuclear Cell (PBMC) Cultures

The PBMC cultures were prepared using 10 mL of venous blood taken from the medial cubital vein of a 23-year-old healthy male volunteer donor who had not consumed alcohol, smoked, or taken any medication that could interfere with the scientific results in the last 72 h. As described in the next section, the number of donors and their characteristics were chosen according to the Organization for Economic Cooperation and Development (OECD) [15]. Blood was collected into a heparin-containing Vacutainer® (approved by the Research Ethics Committee of the Federal University of Pampa, n°. 27045614.0.0000.5323). PBMC were isolated with Histopaque-1077® (Sigma-Aldrich, St. Louis, EUA) and transferred to the culture medium containing 9 mL of RPMI 1640 supplemented with 20% fetal bovine serum and 1% streptomycin/penicillin, as described in previous work [16, 17]. The cells were conditioned in culture flasks and placed in a microenvironment at 37°C in 5% CO_2_ environment for up to 48 hours.

### 2.3. Selection of Concentrations for Tests

Due to a lack of studies on the compound, doses were chosen to allow a broad-spectrum evaluation, which enabled the determination of a median lethal concentration (LC_50_) [16]. Therefore, concentrations of 100 *μ*g/mL, 10 *μ*g/mL, 1 *μ*g/mL, 0.1 *μ*g/mL, and 0.01 *μ*g/mL were initially tested in cultures of PMBC, and, after analysis of cell proliferation, the LC_50_ was determined. The LC_50_ was determined by the statistical method of nonlinear regression. Brazil follows the security assessment protocols proposed by the Organization for Economic Cooperation and Development (OECD). The tests performed here were selected and followed the indications of these protocols for their experimental design when applicable or the indications when suggested. The selected concentrations were based on these OECD indications and relate to decimal fractions of the LC_50_ found.

### 2.4. Treatment of the Cultures

All cultures received Fluazuron diluted in RPMI 1640 in the final volume of 1000 *μ*L. The groups tested were the following: Negative Control (NC) with phosphate buffer pH 7.4, Positive Control (PC) with Colchicine 10 *μ*M, and three concentrations of the acaricide Fluazuron. These concentrations were chosen, as mentioned, based on the LC_50_, as indicated by the OECD. All tests were performed in triplicate. All analyses were performed at time zero, 24 hours, and 48 hours after exposure to Fluazuron.

### 2.5. Effects of Fluazuron on the Cell Viability

The analyzed parameter for evaluation of cytotoxicity was cell viability through the loss of membrane integrity using the trypan blue method [17]. This requires putting the sample in contact with the Trypan blue, which stains dead cells. The analysis was performed using an optical microscope at 400x. One hundred cells were counted.

### 2.6. Genotoxicity Assessment (Alkaline Comet Assay)

This test was performed using the technique described by Singh [18] and Rice-Evans [19]. DNA damage was classified according to the damage index evaluated from the migration of the DNA proteins, which can vary from 0, where there is no damage, until 4, where there is maximum damage. DNA damage was determined as DNA damage index (ID). DNA damage was calculated from cells with different damage classifications; the damage index ranges from 0 (100 cells x 0 when no damage occurred) to 400 (100 cells x 4, when maximum damage occurred).

### 2.7. Mutagenicity Assessment (Micronucleus Test)

The micronucleus test was the parameter used to evaluate mutagenicity. For this, the method described was performed according to description by Schmid [20] and Fenech [21].

### 2.8. Determination of Lymphocyte Subpopulations

Characterization of lymphocyte subpopulations was performed by fluorescence-activated cell sorting (FACS) analysis. The detection of the immune cell fractions was determined using anti-CD45, anti-CD3, anti-CD8, and anti-CD4 antibodies. 15,000 lymphocytes were counted per sample per replicate.

### 2.9. *In Silico* Analyses

In a complementary way and to search for possible methods of action in humans, the compound Fluazuron was submitted to a series of computational tests (*In Silico*) through the platforms: ProTox [8], Way2Drug [10], and GeneCards [11]. The addresses of these platforms are in the references.

### 2.10. Statistical Analysis

All analyses were performed in specific statistical software. Normality distribution analysis was performed by the Kolmogorov-Smirnov test. With the verification that the distribution followed a Gaussian standard, data were evaluated by one-way analysis of variance (ANOVA) followed by Tukey* Post-Hoc* test. Results are expressed as means ±S.D. A p value <0.05 was considered significant. Negative controls and positive presented, in all tests, a statistical difference with p <0.0001.

## 3. Results and Discussion

**Figure 2:**
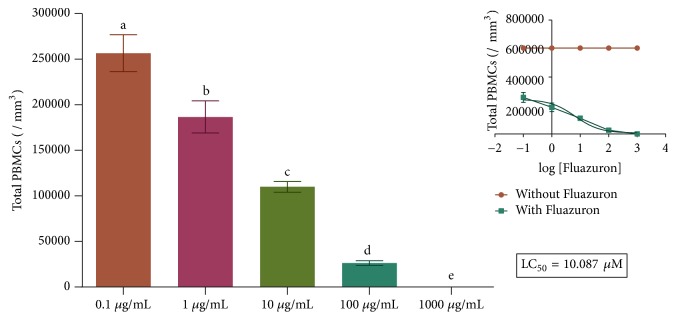
Assessment of cell proliferation for determining the LC_50_ of Fluazuron in PBMC Culture. Inset shows the nonlinear regression curve. Data are expressed as mean ± standard deviation, n=3, performed in triplicate. We considered significant results with p <0.05 for the samples. Different letters mean statistically different values.

As we mentioned, the first protocol aimed at determining the best concentrations to perform the experiment. To do so, we tested a wide curve of Fluazuron concentrations, and the parameter used as standard was cell proliferation, following OECD protocols. The results obtained are shown in Figure 2.

Once the cytotoxicity curve was reached in PBMC, we found that the lethal concentration was close to 10 *μ*M. From this value, the test concentrations for the other protocols were determined as 10, 1, and 0.1 *μ*M.

**Figure 3:**
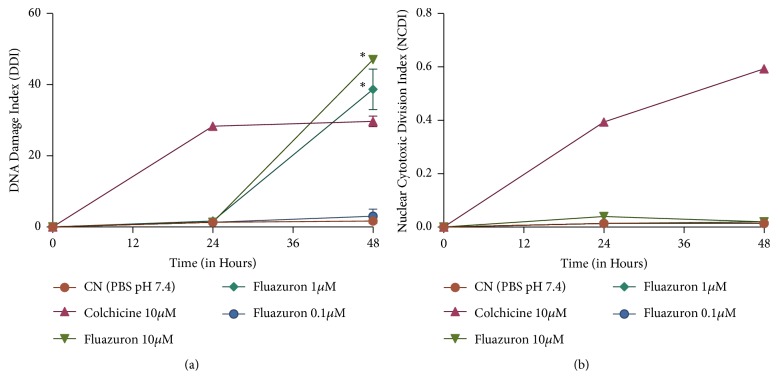
Evaluation of Genotoxicity (a) and Mutagenicity (b) in human PBMC culture, exposed to Fluazuron. Data are expressed as mean ± standard deviation, n=3, performed in triplicate. We considered significant results with p <0.05 for the samples. Different letters mean statistically different values.

**Figure 4:**
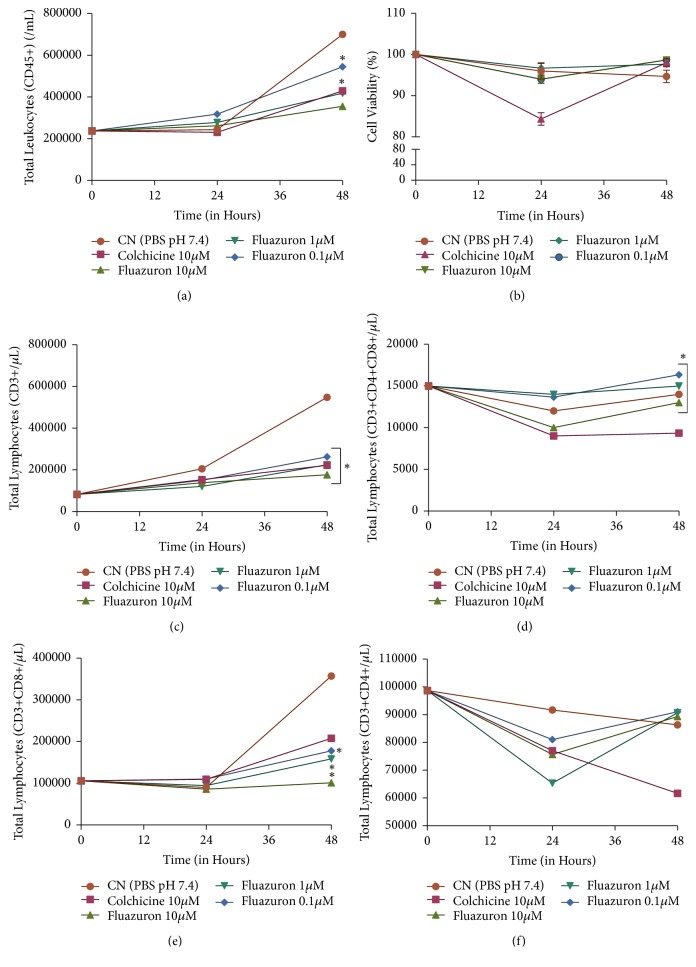
Evaluation of Cytotoxicity of Fluazuron in human PBMC culture. Graph (a) represents the effects on total leukocytes and on (b) the effects on viability. The (c)–(f) graphs show the effects of Fluazuron on lymphocyte subpopulations. Data are expressed as mean ± standard deviation, performed in triplicate. *∗* represents a significant difference in relation to the negative control (p <0.005) in the same contact time.

The other protocols of this experiment were evaluated at three times: initial, 24, and 48 hours after exposure to Fluazuron concentrations. The results are shown in Figures 3 and 4.

As we can see, no concentration tested showed mutagenic effects in the experiment period (p <0.05). The same cannot be said about genotoxic effects. It can be observed in Figure 3(a) that concentrations of 10 and 1 *μ*M cause lesions higher than those caused by the positive control itself in 48 hours of exposure. Although there are no studies that relate the direct effects of Fluazuron on DNA, we can associate this damage with that proposed by the* In Silico* PROTOX platform, which is shown in Table 1.

As we see, the results of Table 1 agree with those found in the experiment, both for mutagenesis and for genotoxicity (carcinogenesis). Protox uses the system of comparison of molecular fractions to analyze its results. This comparison is made with a database of more than 4000 studies of molecules, thus being a very robust and reliable method [22].

Also, chemically Fluazuron is characterized as a 2,6-difluorobenzamide. This molecule has been previously studied and its deleterious effects on DNA are already known. Its action occurs by causing the production of pyknotic nuclei by the disorganized rearrangement of the nuclear chromatin [23].

When analyzing the results of cytotoxicity, we observed that all concentrations caused a decrease in the total growth of leukocytes and lymphocytes (Figures 4(a) and 4(c)), but without reducing the viability of the remaining cells (Figure 4(b)). When we evaluated the lymphocyte subpopulations, we observed that this reduction was due to the specific reduction in the number of CD8 T lymphocytes (cytotoxic) (Figures 4(d)–4(f)).

This is the first time that the effects of Fluazuron immunomodulators are evaluated in lymphocyte subpopulations. Again, we can make use of in silico tools to elucidate the mechanisms involved in this action.  Table 2 shows the results of the evaluation by the Way2Drug Platform associated with Genecards Databank. In these platforms are demonstrated the possible interactions of Fluazuron in biological activities and gene expressions that may be related to the actions verified here.

Inhibition of cytokine production is shown to complement information on the reduction of gamma interferon gene expression. Perhaps this is the most expressive information for the action seen here. The immune system is known to act as a messaging system with multiple exchanges of information along the route. Cytokines are commonly seen as the mediators of these information passages [24]. Without them, the cells are not activated, and the immune response does not continue, which means that there is no need to proliferate the defense cells because there is no defense to be made once the message has been lost [25]. Gamma interferon is one such messenger. Its function is, among others, to activate CD8 T lymphocytes. A reduction in its production leads to a reduction in the continuity of the immune response and to a nonactivation of this subpopulation. With this nonactivation, the cells do not proliferate, and the immune response is weakened, just like the organism [26].

If we consider the information that already postulated that Fluazuron can remain in the body for up to three to four weeks, we are talking about a failure in the immune system that can last up to 30 days, leaving the body totally open to infections and/or unresponsive to pathogens.

Summarizing, Fluazuron can cause genotoxicity by probable chromatin rearrangement and immunodepleting by specific reduction of the CD8 T lymphocyte subpopulation, mediated by the decrease in gamma interferon production. Although the use of Fluazuron is a necessity for tick control and for cattle management, we must bear in mind that the imminent risks to its application exist. Careless use can damage the immune system which in turn carries a gigantic hazard by opening a door to diseases and pathogens and leaving us defenseless. It is up to the health professionals to emphasize the importance of the correct application of the product, as well as the use of protection equipment, but, above all, it is the responsibility of the dissemination of this information.

## Figures and Tables

**Table 1:** Computational analysis of probable toxicity mechanisms for Fluazuron using the PROTOX-II Platform [8].

Target	Prediction	Probability
*Hepatotoxicity*	*Active*	*0.72*
*Carcinogenicity*	*Active*	*0.56*
*Immunotoxicity*	*Active*	*0.55*
Mutagenicity	Inactive	0.93
*Cytotoxicity*	*Active*	*0.51*
Aryl hydrocarbon Receptor (AhR)	Inactive	0.90
Androgen Receptor (AR)	Inactive	1.0
Androgen Receptor Ligand Binding Domain (AR-LBD)	Inactive	0.99
Aromatase	Inactive	0.97
Estrogen Receptor Alpha (ER)	Inactive	0.98
Estrogen Receptor Ligand Binding Domain (ER-LBD)	Inactive	1.0
Peroxisome Proliferator Activated Receptor Gamma	Inactive	0.78
Heat shock factor response element (HSE)	Inactive	0.97
*Mitochondrial Membrane Potential (MMP)*	*Active*	*1.0*
*Phosphoprotein (Tumor Suppressor) p53*	*Active*	*1.0*
ATPase family AAA domain-containing protein 5	Inactive	0.99

**Table 2:** Computational analysis of probable biological interactions for Fluazuron using the Way2Drug Platform [10] Associated with Genecards Databank [11].

Target	Prediction	Genecards information
Cytokine production inhibitor	Active	-* *-* *-* *-* *-* *-
DNA polymerase I inhibitor	Active	-* *-* *-* *-* *-* *-
Increases expression of the ATG5 gene	Active	Protein involved in the formation of the Autophagic Vesicle in Cell Death.
Reduces expression of the CCNC gene	Active	Cyclin protein involved in apoptosis routes.
Reduces IFNG gene expression	Active	*γ* Interferon protein, important mediator of the immune system.

## Data Availability

The data used to support the findings of this study are available from the corresponding author upon request.
